# 3-Methyl­thio­benzamide

**DOI:** 10.1107/S1600536809019849

**Published:** 2009-05-29

**Authors:** Mahmood-ul-Hassan Khan, Shahid Hameed, Tashfeen Akhtar, Jason D. Masuda

**Affiliations:** a Department of Chemistry, Quaid-i-Azam University, Islamabad-45320, Pakistan; b Department of Chemistry, Saint Mary’s University, Halifax, Nova Scotia, Canada B3H 3C3

## Abstract

In the title compound, C_8_H_9_NS, the dihedral angle between the aromatic ring and the thio­amide fragment is 36.0 (2)°. There are π-stacking inter­actions between coplanar aryl fragments, with a centroid–centroid separation of 3.658 (2) Å. In addition, there are inter­molecular hydrogen bonds between the amino group and the S atoms.

## Related literature

For our previous work on the synthesis and biological screening of five-membered heterocycles, see: Akhtar *et al.* (2006 ▶, 2007 ▶, 2008 ▶); Serwar *et al.* (2009 ▶). For related structures, see: Jian *et al.* (2006 ▶); Khan *et al.* (2009*a* ▶,*b* ▶).
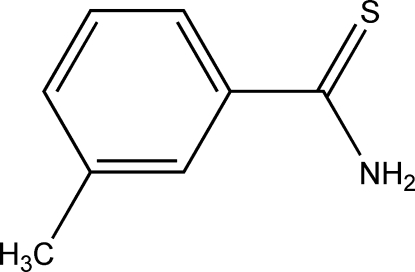


## Experimental

### 

#### Crystal data

C_8_H_9_NS
*M* *_r_* = 151.22
Monoclinic, 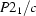

*a* = 7.717 (5) Å
*b* = 10.267 (7) Å
*c* = 10.100 (7) Å
β = 97.186 (9)°
*V* = 794.0 (9) Å^3^
*Z* = 4
Mo *K*α radiation
μ = 0.33 mm^−1^
*T* = 296 K
0.37 × 0.27 × 0.20 mm

#### Data collection

Bruker APEXII CCD diffractometer
Absorption correction: multi-scan (*SADABS*; Bruker, 2008 ▶) *T*_min_ = 0.793, *T*_max_ = 0.930
6234 measured reflections
1797 independent reflections
1447 reflections with *I* > 2σ(*I*)
*R*_int_ = 0.021

#### Refinement

*R*[*F* ^2^ > 2σ(*F* ^2^)] = 0.039
*wR*(*F* ^2^) = 0.112
*S* = 1.08
1797 reflections
92 parameters
H-atom parameters constrained
Δρ_max_ = 0.22 e Å^−3^
Δρ_min_ = −0.29 e Å^−3^

### 

Data collection: *APEX2* (Bruker, 2008 ▶); cell refinement: *SAINT* (Bruker, 2008 ▶); data reduction: *SAINT*; program(s) used to solve structure: *SHELXS97* (Sheldrick, 2008 ▶); program(s) used to refine structure: *SHELXL97* (Sheldrick, 2008 ▶); molecular graphics: *ORTEP-3 for Windows* (Farrugia, 1997 ▶); software used to prepare material for publication: *SHELXTL* (Sheldrick, 2008 ▶).

## Supplementary Material

Crystal structure: contains datablocks I, global. DOI: 10.1107/S1600536809019849/bt2967sup1.cif

Structure factors: contains datablocks I. DOI: 10.1107/S1600536809019849/bt2967Isup2.hkl

Additional supplementary materials:  crystallographic information; 3D view; checkCIF report

## Figures and Tables

**Table 1:** Hydrogen-bond geometry (Å, °)

*D*—H⋯*A*	*D*—H	H⋯*A*	*D*⋯*A*	*D*—H⋯*A*
N1—H1*A*⋯S1^i^	0.86	2.66	3.455 (2)	155
N1—H1*B*⋯S1^ii^	0.86	2.58	3.422 (3)	165

Symmetry codes: (i) 
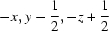
; (ii) 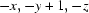.

**Fig. 1.**
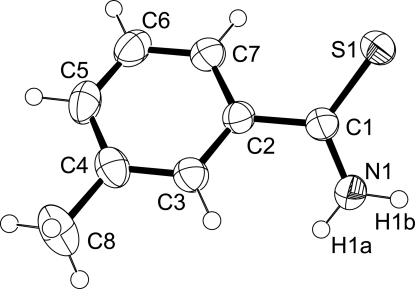
Molecular structure of 3-methylthiobenzamide showing displacement ellipsoids at the 50% probability level (for non-H atoms).

**Fig. 2.**
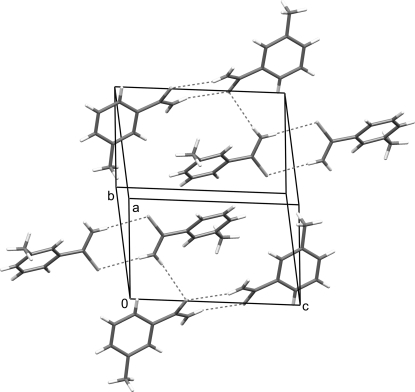
Packing diagram of 3-methylthiobenzamide. Hydrogen bonds shown as dashed lines.

## supplementary crystallographic information

### Comment

Thioamides are not only important intermediates in the synthesis of heterocyclic compounds but they also possess enormous biologically activities as reported in our previous articles (Khan *et al.*, 2009a). In the present article, we report the crystal structure of 3-methylthiobenzamide, synthesized as a continuation of our previous work on the synthesis and biological screenings of five membered heterocycles (Akhtar *et al.*, 2006, 2007, 2008; Serwar *et al.*, 2009).

There are two distinct hydrogen bonding interactions between the nitrogen and sulfur atoms. The first arranges the dimer with N···S distances of 3.422 (3)Å and the second links two thioamide dimers through another N···S interaction on the order of 3.455 (2) Å. These N—H···S hydrogen bonding interactions are similar to those seen in *p*-trifluoromethylbenzothioamide where the corresponding interactions are between 3.3735Å and 3.5133Å (Jian *et al.*, 2006), in 4-chlorobenzothioamide where the N···S distances are 3.3769 (15)Å and 3.4527 (15)Å (Khan *et al.*, 2009a) and in 4-bromobenzothioamide where the N···S distances are between 3.500 (2)Å and 3.605 (3) Å (Khan *et al.*, 2009b).

### Experimental

The title compound was synthesized from 3-methylbenzonitrile according to a reported procedure (Khan *et al.*, 2009a). The recrystallization of the product from chloroform afforded   crystals suitable for X-ray analysis.

### Refinement

The hydrogen atoms were placed in geometrically idealized positions with C—H distances of 0.93Å (aromatic C—H), 0.96Å (methyl) and 0.86Å (amide N—H) and constrained to ride on the parent atom with *U*_iso_(H) = 1.2 U_eq_(*C*) for aromatic and amide protons or *U*_iso_(H) = 1.5 U_eq_(*C*_methyl_).

### Crystal data

**Table:**

C_8_H_9_NS	*F*(000) = 320
*M**_r_* = 151.22	*D*_x_ = 1.265 Mg m^−^^3^
Monoclinic, *P*2_1_/*c*	Mo *K*α radiation, λ = 0.71073 Å
Hall symbol: -P 2ybc	Cell parameters from 2572 reflections
*a* = 7.717 (5) Å	θ = 2.7–27.1°
*b* = 10.267 (7) Å	µ = 0.33 mm^−^^1^
*c* = 10.100 (7) Å	*T* = 296 K
β = 97.186 (9)°	Block, yellow
*V* = 794.0 (9) Å^3^	0.37 × 0.27 × 0.20 mm
*Z* = 4	

### Data collection

**Table:**

Bruker APEXII CCD diffractometer	1797 independent reflections
Radiation source: fine-focus sealed tube	1447 reflections with *I* > 2σ(*I*)
graphite	*R*_int_ = 0.021
φ and ω scans	θ_max_ = 27.5°, θ_min_ = 2.7°
Absorption correction: multi-scan (*SADABS*; Bruker, 2008)	*h* = −10→9
*T*_min_ = 0.793, *T*_max_ = 0.930	*k* = −13→13
6234 measured reflections	*l* = −11→13

### Refinement

**Table:**

Refinement on *F*^2^	Primary atom site location: structure-invariant direct methods
Least-squares matrix: full	Secondary atom site location: difference Fourier map
*R*[*F*^2^ > 2σ(*F*^2^)] = 0.039	Hydrogen site location: inferred from neighbouring sites
*wR*(*F*^2^) = 0.112	H-atom parameters constrained
*S* = 1.08	*w* = 1/[σ^2^(*F*_o_^2^) + (0.0556*P*)^2^ + 0.242*P*] where *P* = (*F*_o_^2^ + 2*F*_c_^2^)/3
1797 reflections	(Δ/σ)_max_ < 0.001
92 parameters	Δρ_max_ = 0.22 e Å^−^^3^
0 restraints	Δρ_min_ = −0.29 e Å^−^^3^

### Special details

**Table:**

Geometry. All e.s.d.'s (except the e.s.d. in the dihedral angle between two l.s. planes) are estimated using the full covariance matrix. The cell e.s.d.'s are taken into account individually in the estimation of e.s.d.'s in distances, angles and torsion angles; correlations between e.s.d.'s in cell parameters are only used when they are defined by crystal symmetry. An approximate (isotropic) treatment of cell e.s.d.'s is used for estimating e.s.d.'s involving l.s. planes.
Refinement. Refinement of *F*^2^ against ALL reflections. The weighted *R*-factor *wR* and goodness of fit *S* are based on *F*^2^, conventional *R*-factors *R* are based on *F*, with *F* set to zero for negative *F*^2^. The threshold expression of *F*^2^ > σ(*F*^2^) is used only for calculating *R*-factors(gt) *etc*. and is not relevant to the choice of reflections for refinement. *R*-factors based on *F*^2^ are statistically about twice as large as those based on *F*, and *R*- factors based on ALL data will be even larger.

### Fractional atomic coordinates and isotropic or equivalent isotropic displacement parameters (Å^2^)

**Table:**

	*x*	*y*	*z*	*U*_iso_*/*U*_eq_	
S1	0.13324 (7)	0.62675 (4)	0.16035 (4)	0.05023 (19)	
N1	0.0243 (2)	0.38549 (15)	0.17149 (16)	0.0521 (4)	
H1A	0.0187	0.3113	0.2103	0.063*	
H1B	−0.0260	0.3963	0.0914	0.063*	
C2	0.1933 (2)	0.45618 (15)	0.37261 (16)	0.0358 (4)	
C1	0.1099 (2)	0.48241 (16)	0.23470 (16)	0.0379 (4)	
C3	0.2661 (2)	0.33439 (17)	0.40481 (18)	0.0406 (4)	
H3A	0.2535	0.2683	0.3414	0.049*	
C7	0.2072 (2)	0.55347 (17)	0.46931 (18)	0.0435 (4)	
H7A	0.1589	0.6352	0.4492	0.052*	
C4	0.3569 (2)	0.30980 (18)	0.52939 (19)	0.0455 (4)	
C5	0.3695 (2)	0.4084 (2)	0.62387 (18)	0.0500 (5)	
H5A	0.4303	0.3935	0.7079	0.060*	
C6	0.2927 (3)	0.5286 (2)	0.59491 (19)	0.0501 (5)	
H6A	0.2989	0.5927	0.6604	0.060*	
C8	0.4466 (3)	0.1803 (2)	0.5575 (2)	0.0671 (6)	
H8A	0.3710	0.1115	0.5210	0.101*	
H8B	0.4729	0.1686	0.6522	0.101*	
H8C	0.5529	0.1786	0.5173	0.101*	

### Atomic displacement parameters (Å^2^)

**Table:**

	*U*^11^	*U*^22^	*U*^33^	*U*^12^	*U*^13^	*U*^23^
S1	0.0774 (4)	0.0330 (2)	0.0387 (3)	0.0000 (2)	0.0007 (2)	0.00264 (17)
N1	0.0726 (11)	0.0435 (8)	0.0363 (8)	−0.0142 (7)	−0.0089 (7)	0.0048 (6)
C2	0.0384 (8)	0.0362 (8)	0.0329 (8)	−0.0031 (6)	0.0046 (6)	0.0001 (6)
C1	0.0439 (9)	0.0345 (8)	0.0351 (9)	0.0016 (6)	0.0042 (7)	−0.0013 (6)
C3	0.0461 (9)	0.0380 (8)	0.0379 (9)	−0.0003 (7)	0.0066 (7)	0.0007 (7)
C7	0.0508 (10)	0.0396 (9)	0.0398 (9)	−0.0018 (7)	0.0045 (8)	−0.0032 (7)
C4	0.0433 (9)	0.0492 (10)	0.0446 (10)	0.0012 (7)	0.0078 (7)	0.0129 (8)
C5	0.0482 (10)	0.0674 (12)	0.0332 (9)	−0.0082 (9)	−0.0003 (8)	0.0076 (8)
C6	0.0576 (11)	0.0554 (11)	0.0369 (10)	−0.0097 (9)	0.0040 (8)	−0.0083 (8)
C8	0.0737 (14)	0.0647 (14)	0.0630 (14)	0.0205 (11)	0.0093 (11)	0.0223 (11)

### Geometric parameters (Å, °)

**Table:**

S1—C1	1.6811 (19)	C7—H7A	0.9300
N1—C1	1.315 (2)	C4—C5	1.386 (3)
N1—H1A	0.8600	C4—C8	1.509 (3)
N1—H1B	0.8600	C5—C6	1.384 (3)
C2—C7	1.392 (2)	C5—H5A	0.9300
C2—C3	1.393 (2)	C6—H6A	0.9300
C2—C1	1.484 (2)	C8—H8A	0.9600
C3—C4	1.385 (3)	C8—H8B	0.9600
C3—H3A	0.9300	C8—H8C	0.9600
C7—C6	1.379 (3)		
			
C1—N1—H1A	120.0	C3—C4—C5	118.45 (17)
C1—N1—H1B	120.0	C3—C4—C8	119.99 (18)
H1A—N1—H1B	120.0	C5—C4—C8	121.49 (19)
C7—C2—C3	119.16 (16)	C6—C5—C4	120.95 (18)
C7—C2—C1	120.98 (15)	C6—C5—H5A	119.5
C3—C2—C1	119.80 (15)	C4—C5—H5A	119.5
N1—C1—C2	116.80 (15)	C7—C6—C5	120.17 (17)
N1—C1—S1	121.71 (14)	C7—C6—H6A	119.9
C2—C1—S1	121.41 (12)	C5—C6—H6A	119.9
C4—C3—C2	121.29 (17)	C4—C8—H8A	109.5
C4—C3—H3A	119.4	C4—C8—H8B	109.5
C2—C3—H3A	119.4	H8A—C8—H8B	109.5
C6—C7—C2	119.91 (17)	C4—C8—H8C	109.5
C6—C7—H7A	120.0	H8A—C8—H8C	109.5
C2—C7—H7A	120.0	H8B—C8—H8C	109.5
			
N1—C1—C2—C3	36.0 (2)		

### Hydrogen-bond geometry (Å, °)

**Table:**

*D*—H···*A*	*D*—H	H···*A*	*D*···*A*	*D*—H···*A*
N1—H1A···S1^i^	0.86	2.66	3.455 (2)	155
N1—H1B···S1^ii^	0.86	2.58	3.422 (3)	165

Symmetry codes: (i) −*x*, *y*−1/2, −*z*+1/2; (ii) −*x*, −*y*+1, −*z*.
